# The Characterization and Identification of Cyperus Protein: An In Vitro Study on Its Antioxidant and Anti-Inflammatory Potential

**DOI:** 10.3390/nu17162633

**Published:** 2025-08-14

**Authors:** Qian Zhang, Chaoyue Ma, Xiaotong Wu, Huifang Hao

**Affiliations:** School of Life Sciences, Inner Mongolia University, Hohhot 010070, China; 18734309982@163.com (Q.Z.); mcy32108087@163.com (C.M.)

**Keywords:** cyperus protein, antioxidant peptide, non-targeted metabolomics, molecular docking, in vitro experiments

## Abstract

**Background:** Oxidative stress and inflammation are major drivers of metabolic inflammatory diseases, and natural antioxidant peptides represent promising therapeutic agents. Antioxidant peptides derived from Cyperus protein (CAOP) exhibit high digestibility and bioavailability, but their antioxidant and anti-inflammatory mechanisms remain unclear. **Methods:** We employed in vitro experiments, non-targeted metabolomics, peptide omics, and molecular docking techniques to explore how CAOP exerts dual antioxidant and anti-inflammatory effects. **Results:** The in vitro experiments showed that in LPS-induced RAW264.7 cells, CAOP not only significantly increased the levels of superoxide dismutase (SOD) and catalase (CAT) but also significantly reduced the gene expression and secretion of interleukin-6 (IL-6) and tumor necrosis factor-α (TNF-α), as well as the phagocytic ability of cells. Metabolomics studies indicate that CAOP protects cells from LPS-induced damage by enhancing intracellular glutathione metabolism pathways, glyceraldehyde and dicarboxylic acid metabolism pathways, pantothenic acid and coenzyme A biosynthesis metabolism pathways, and thiamine metabolism pathways while inhibiting the ferroptosis pathway. CAOP was purified using Sephadex G-25 column chromatography, and its amino acid sequence was determined using LC-MS/MS technology. Subsequently, 25 peptide sequences were screened through bioinformatics analysis. These peptides can target Keap1. Among them, DLHMFVWS (-ICE = 62.8072) and LGHPWGNAPG (-ICE = 57.4345) are most likely to activate the Nrf2-Keap1 pathway. **Conclusions:** CAOP exerts antioxidant and anti-inflammatory effects by regulating the key metabolic networks, demonstrating its therapeutic promise for associated with oxidative damage and metabolic inflammation disorders.

## 1. Introduction

Oxidative stress is a core pathological driver of metabolic inflammatory diseases such as Alzheimer’s disease [1], traumatic brain injury [2], chronic obstructive pulmonary disease [3], diabetes [4], cardiovascular disease [5], and cancer [6]. Antioxidants are primary therapeutic agents that can alleviate oxidative stress, maintain bodily health, and potentially prevent disease onset [7]. However, synthetic antioxidants pose potential safety risks, including toxicity and carcinogenicity [8]. Natural antioxidant peptides have emerged as superior alternatives due to their higher safety profile, bioavailability, and multi-target regulatory potential.

Cyperus (*Cyperus esculentus* L.) is an edible perennial grass-like plant native to the Nile River basin, now widely cultivated worldwide [9]. It is rich in nutrients, including oils, starch, dietary fiber, protein, flavonoids, and phenolic compounds. Its unique phytochemical composition gives it significant potential in the fields of functional foods and pharmaceuticals [10]. Research has confirmed its multifaceted bioactive properties, including anti-tumor, antioxidant, anti-thrombotic, and antibacterial capabilities [11]. Notably, it has protective effects on the nervous system [12] and cardiovascular system [13], and it holds promise for preventing diabetes [14] and gastrointestinal diseases [15]. Its confirmed therapeutic applications include the treatment of chronic gastritis, lymphatic tuberculosis, heat injuries, coronary heart disease, acute cholecystitis, and acute cerebral hemorrhage [16]. Cyperus protein is characterized by its high quality and excellent digestibility, with 70.59% of the protein being susceptible to pepsin digestion [17]. Fang et al. have documented an essential amino acid index of 93.8 and a biological value of 90.54 for Cyperus protein, surpassing soy protein and closely approaching egg protein values [18]. Yin Haiyang et al. successfully isolated a peptide from Cyperus that exhibited potent ACE inhibitory properties [19], while Yang Zhuo identified an ACE-inhibiting peptide through the addition of alkaline protease to Cyperus protein hydrolysate [20]. Additionally, Ma et al. demonstrated the ability of the Cyperus peptide SFRWQ to enhance RAW264.7 cell resilience against LPS-induced injury [21]. Current research on Cyperus has mainly focused on protein extraction and oil preparation. However, there are limited reports on the molecular mechanisms of antioxidant and anti-inflammatory effects of antioxidant peptides derived from Cyperus protein, which has to some extent restricted the development and utilization of Cyperus protein.

This study pioneered the use of a multi-omics integration approach, combining peptidomics, metabolomics, and bioinformatics, to preliminarily reveal the antioxidant and anti-inflammatory mechanisms of Cyperus protein (CAOP). These findings create more opportunities for CAOP as a therapeutic candidate for metabolic inflammatory diseases and promote the sustainable utilization of Cyperus by-products.

## 2. Materials and Methods

### 2.1. Cell Experiment

#### 2.1.1. Cell Culture

The RAW264.7 murine macrophage cell line was provided by our institutional laboratory. The RAW264.7 cells were cultured in DMEM (containing 10% fetal bovine serum and 1% penicillin–streptozotocin) at 37 °C in a humidified incubator with 5% CO_2_.

#### 2.1.2. Cell Cytotoxicity Assay

The CAOP was prepared according to the existing conditions in the laboratory [22]. RAW264.7 cells were seeded on a 96-well microplate at a density of 1 × 10^4^ cells/well and cultured for 24 h and then treated with different concentrations (5–80 mg/mL) of the CAOP for another 24 h. Cell cytotoxicity was detected by the MTT (Mreda, Beijing, China) method.

#### 2.1.3. Determination of Neutral Red Phagocytosis 

Cells were plated in 96-well plates (1.0 × 10^4^ cells/well), exposed to 10, 20 and 40 mg/mL CAOP for 2 h, and subsequently challenged with 100 ng/mL LPS (Sigma, City of Saint Louis, MO, USA) for 24 h. Finally, the cells were treated with 0.05% neutral red solution for 2 h, after which the absorbance was measured at 540 nm.

#### 2.1.4. Determination of Intracellular SOD and CAT Activities

Cell processing as in Section 2.1.3. Determination of superoxide dismutase (SOD) and catalase (CAT) (Geryisi, Shanghai, China) activities was carried out using kits according to manufacturer’s instructions (Geryisi). The cultured cells were collected and crushed on ice with an ultrasonic breaker. The cleaved cells were centrifuged at 4 °C at 10,000× *g* for 10 min, and total protein concentration in the supernatant was determined using a BCA protein analysis kit (Elabscience Biotechnology Co., Ltd. (Wuhan, China)). The contents of SOD and CAT were expressed as a ratio to total protein.

#### 2.1.5. Measurement of IL-6 and TNF-α Gene Expression

Cell processing as in Section 2.1.3. SYBR Green real-time polymerase chain reaction (Youyi Landi Biotechnology Co., Ltd. (Suzhou, China)) was used to detect the transcription level of target gene mRNA in cell samples. Data were standardized by comparison with actin. The three pairs of primers used in this experiment, β-actin (Beta-actin), TNF-α (Tumor Necrosis Factor-alpha), and IL-6 (Interleukin-6), are the same as those used in the study published by Ma et al. from the same laboratory [21].

#### 2.1.6. IL-6 and TNFα Quantification

Cell processing was the same as in Section 2.1.3. Collect cell culture supernatant and use an ELISA (Enzyme-linked immunosorbent assay) kit to determine the levels of IL-6 and TNFα (Neobioscience, Shanghai, China).

### 2.2. Non-Targeted Metabolomics

#### 2.2.1. Sample Preparation

RAW264.7 macrophages were cultured in 10 cm dishes for 24 h. The PL group received 2 h exposure to 10 mg/mL CAOP prior to a 24 h stimulation with 100 ng/mL LPS, while the LPS group was treated directly with 100 ng/mL LPS for 24 h. Cell pellets were resuspended in an ice-cold solvent mixture (methanol/acetonitrile/water, 2:2:1 volume ratio), sonicated on ice, stored at −20 °C for 1 h, and the supernatant was lyophilized and redissolved in 150 μL of acetonitrile/water (1:1 volume ratio). QC samples were composed of 10 μL mixed samples from each experimental group.

#### 2.2.2. LC–MS/MS Analysis

The operating procedures for LC-MS/MS are the same as those described in the study published by Ma et al. [21] in the same laboratory.

### 2.3. Isolation, Purification and Structure Identification of CAOP

#### 2.3.1. Sephadex G-25 Medium

The injection valve was equilibrated with deionized water prior to sample loading. CAOP (5 mg/mL) was purified and eluted isocratically on a Sephadex G-25 column (Runcheng Biotechnology, Co., Ltd. (Shanghai, China)) using a 2 mL loading volume and ultrapure water at a flow rate of 2 mL/min. The change in the absorbance value at 280 nm was measured in real time, and each 5 mL of eluate was used as a fraction until the separation was terminated when the absorbance value at 280 nm was 0 and remained constant.

#### 2.3.2. UPLC–MS/MS-Based Peptide Identification

The fractionated components were dissolved in deionized water (ddH_2_O). The sample was loaded onto a C18 column (150 μm × 150 mm) at a flow rate of 600 nL/min, using a 120-min linear gradient elution (gradient program: see Table 1). The mobile phase composition is as follows: A—an aqueous solution containing 0.1% (*v*/*v*) formic acid, and B—a mixed solvent containing 80% acetonitrile, 0.1% formic acid, and 20% water (*v*/*v*). After data acquisition is completed, target compound analysis is performed.

### 2.4. CAOP Component Screening and Structure–Activity Relationships

#### 2.4.1. Structure Activity Relationship Analysis of Peptides

This study used density functional theory (DFT) for calculations. The software used in this process was GaussView 5.0 and Gaussian 09. Molecular mechanics MM^+^ was utilized for the subsequent refinement of the conformational geometry. Subsequently, in Gaussian 09, energy minimization calculations were performed on the peptide segment using the B3LYP/6-31G** basis set. Electronic properties, including EHOMO, ELUMO and molecular energy, were calculated using quantum chemical methods.

#### 2.4.2. Molecular Docking

We performed molecular docking simulations using CDOCKER (Discovery Studio 2019). The crystallographic structure of human Keap1 (PDB: 2FUL) was preprocessed for docking simulations. TX6 (PubChem CID: 121488089) was selected as the benchmark ligand due to its high-affinity binding to Keap1 and activation of the Keap1-Nrf2 pathway [14]. Ligand screening employed peptide sequence libraries. During docking, the Keap1 receptor remained constrained as rigid, whereas both TX6 and peptide segments retained conformational flexibility. We evaluated molecular docking results through CDOCKER interaction energy (-CIE) scores, interaction sites, and interaction types.

### 2.5. Metabolomics and Bioinformatics Analysis

Chromatographic peak extraction was conducted employing R software (v4.2.2) and material identification was performed via MetaboAnalyst 5.0 (https://www.metaboanalyst.ca/, accessed on 8 July 2023), while dataset processing utilized the Luming (https://cloud.oebiotech.cn/task/, accessed on 13 July 2023) and Lianchuan Ata (http://www.lc-bio.com/, accessed on 30 July 2023) cloud-based platforms.

### 2.6. Statistical Analysis

Statistical analysis was performed using GraphPad Prism 9 software. All experiments were performed at least three times independently, and the results are expressed as the mean ± standard deviation. Statistical comparisons were performed using *t*-tests or a one-way analysis of variance (ANOVA).

## 3. Results

### 3.1. Cell Viability

**Figure 1:**
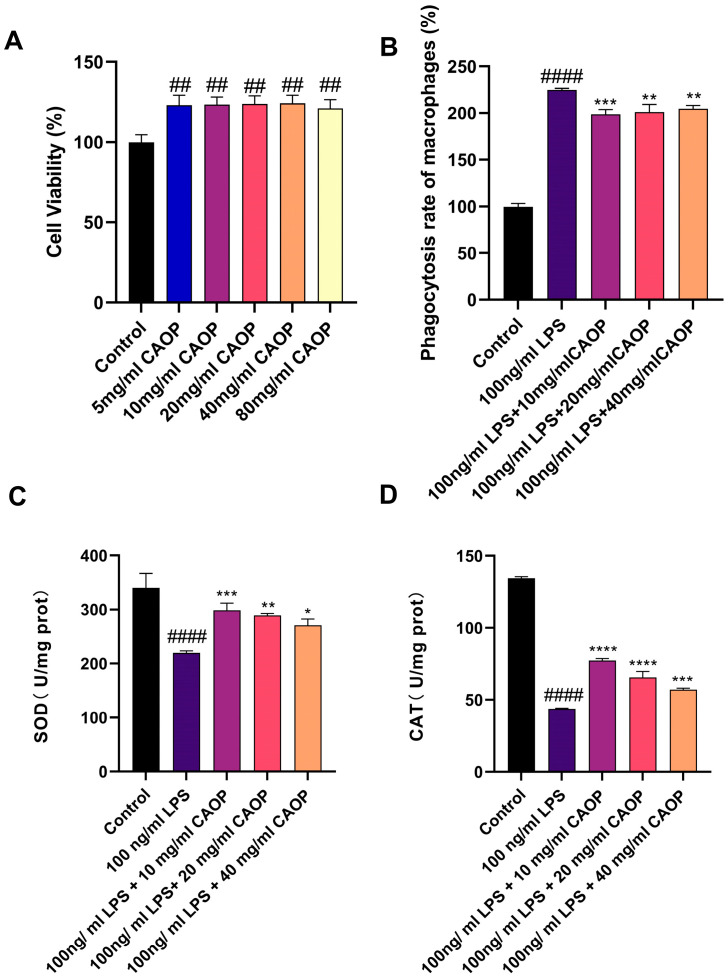
(**A**) Cell activity; (**B**) phagocytosis rate of macrophages; (**C**) SOD; (**D**) CAT. ^##^ *p* <0.01, ^####^ *p* < 0.0001 compared with the control group; * *p* < 0.05, ** *p* <0.01, *** *p* < 0.001, **** *p* < 0.0001 compared with the injury group.

According to previous studies [23], peptides are considered non-cytotoxic when the cell survival rate after treatment exceeds 80%. CAOP was non-cytotoxic to RAW264.7 macrophages at concentrations ranging from 5 to 80 mg/mL. Furthermore, it enhanced cell survival rates across the entire dose range (Figure 1A).

### 3.2. Neutral Red Phagocytosis Assay

RAW264.7 cells transformed into M1 macrophages after exogenous stimulation (LPS), and their phagocytic function was significantly enhanced. A neutral red phagocytosis test showed that the phagocytic ability of cells in the LPS group was greatly increased. CAOP treatment inhibited phagocytosis compared to the LPS group, indicating that it can inhibit the stimulatory effect of LPS on RAW264.7 cells (Figure 1B).

### 3.3. The Effect of CAOP on Cell SOD and MDA Content

The antioxidant efficacy of CAOP in oxidatively stressed RAW264.7 macrophages was assessed by measuring SOD and CAT activities. As shown in Figure 1C,D, LPS significantly inhibited the activity of both enzymes compared to the control group. CAOP intervention reversed this inhibitory effect and alleviated LPS-induced oxidative damage.

### 3.4. Expression of Inflammatory Factors in RAW264.7 Cells

**Figure 2:**
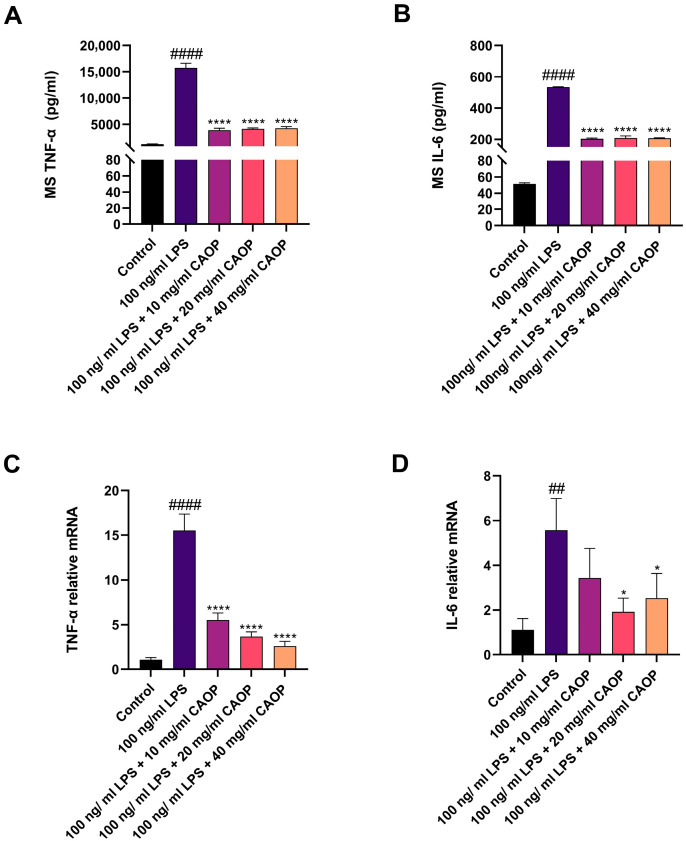
(**A**) TNF-α secretion; (**B**) IL-6 secretion; (**C**) TNF-α mRNA; (**D**) IL-6 mRNA. ^##^ *p* <0.01, ^####^ *p* < 0.0001 compared with the control group; * *p* <0.05, **** *p* < 0.0001 compared with the injury group.

Illustrated in Figure 2A,B, CAOP (10, 20, or 40 mg/mL) demonstrates a concentration-graded attenuation of LPS-provoked elevations in IL-6 and TNF-α. To delineate the underlying anti-inflammatory mechanisms, the transcription levels of inflammatory factors were quantified. As depicted in Figure 2C,D, LPS stimulation significantly upregulated IL-6 and TNF-α mRNA expression, whereas pretreatment with CAOP reduced these effects in RAW264.7 macrophages.

### 3.5. Metabolomics Analysis

**Figure 3:**
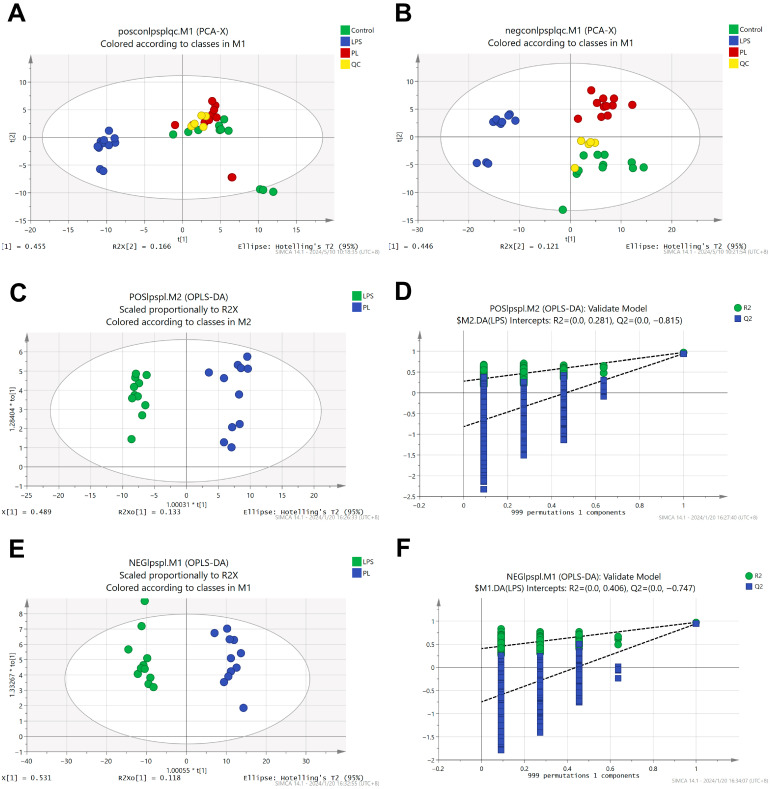
(**A**) Global metabolome of positive mode principal component analysis (PCA). (**B**) Negative mode principal component analysis (PCA). (**C**) OPLS-DA of PL and LPS in positive mode. (**D**) Permutation test in positive mode. (**E**) OPLS-DA of PL and LPS in negative mode. (**F**) Permutation test in negative mode.

**Figure 4:**
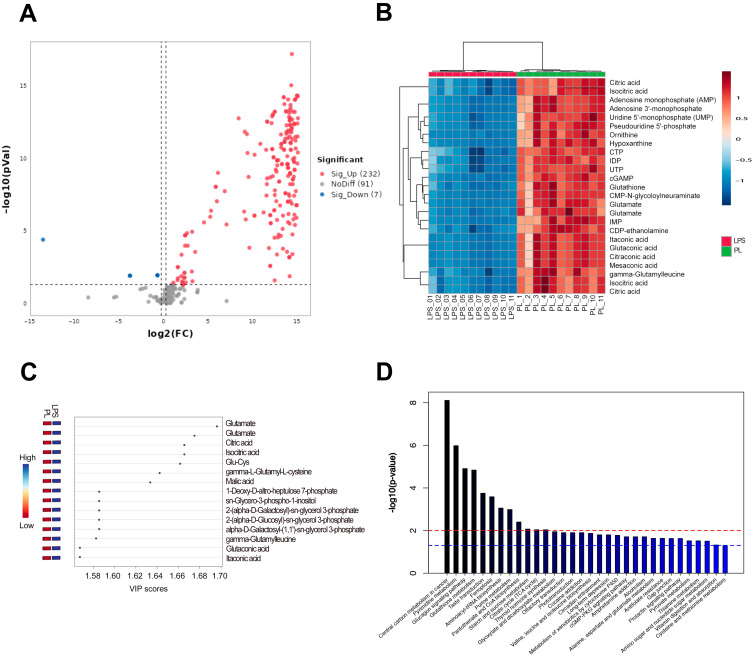
(**A**) Volcano plot. (**B**) Heatmap of differentially abundant metabolites. (**C**) VIP score analysis. (**D**) KEGG enrichment analysis of differential metabolites.

Non-targeted metabolomics analysis identified 330 metabolites. Multivariate analysis was performed on these metabolite profiles, and quality control samples were intermittently inserted to verify system stability. Principal component analysis (PCA) showed significant separation between the LPS group and the PL group in cationic and anionic metabolites, while the close clustering of quality control samples indicated the reliability and reproducibility of the method (Figure 3A,B). OPLS-DA modeling assessed cationic and anionic substance alignment without overfitting (Figure 3C–F), confirming experimental grouping validity. Finally, volcano plot analysis identified 229 metabolites with altered abundance between the LPS group and the PL group. Among them, 223 metabolites were upregulated, and 7 were downregulated (log_2_FC > 1.2, *p* < 0.05) (Figure 4A).

#### 3.5.1. Differentially Abundant Metabolite Analysis

A cluster analysis of the top 25 metabolites with significant abundance changes (Figure 4B) showed the abundance of hypoxanthine, inosine 5′-diphosphate, glutathione, CDP-ethanolamine, itaconic acid, succinic acid, citric acid, methyl citric acid, γ-glutamyl derivatives, leucine, isocitric acid, and citric acid. After CAOP pretreatment followed by LPS treatment, the abundance of these metabolites was significantly increased compared to LPS exposure alone. The cluster analysis indicated no significant differences within groups.

Differential metabolites were screened using VIP > 1.00 and *p* < 0.05. Figure 4C shows the VIP scores, which measure the contribution of each metabolite to the classification or predictive ability of the model. Metabolites with VIP > 1.5 were considered to significantly contribute to the clustering of the LPS group and the peptide-pretreated group, including glutamic acid, citric acid, isocitric acid, glutathione, γ-L-glutamyl-L-cysteine, malic acid, Sn-glycerol-3-phosphate-1-inositol, γ-glutamyl-leucine, and glutaric acid.

#### 3.5.2. Pathways Related to the Differentially Abundant Metabolites

Metabolic pathway enrichment analysis mapped 229 metabolites with altered abundance to 110 KEGG pathways. Figure 4D shows the top 20 enriched pathways, mainly involving amino acid metabolism, cofactor and vitamin metabolism, and cell growth and death regulation. The metabolic process of amino acids mainly refers to the biosynthesis of glutathione and arginine and the biosynthesis of valine, leucine and isoleucine. Cell growth and death mainly include ferroptosis. The metabolic process of cofactors and vitamins mainly refers to the metabolism of thiamine, the production of pantothenic acid, and the biosynthesis of coenzyme a. Carbohydrate processing encompasses the tricarboxylic acid (TCA) cycle, glyoxylate–dicarboxylate processing, and starch–sucrose catabolism.

### 3.6. Results of Sephadex G-25 Chromatography

**Figure 5:**
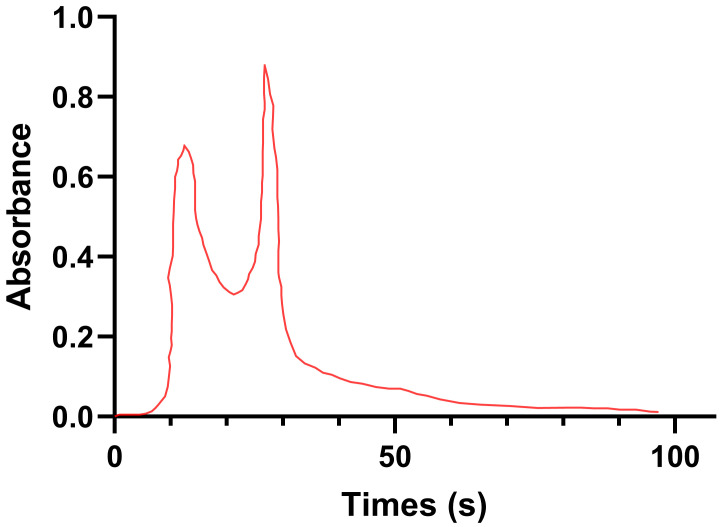
Sephadex G-25 gel chromatography chromatogram of aqueous CAOP.

A chromatogram is shown in Figure 5. CAOP yielded two chromatographic peaks (fraction a and fraction b) following purification on a Sephadex G-25 gel column, and the content of CAOP was 44.77% (fraction a) and 55.23% (fraction b), respectively. DPPH· clearance was determined for fraction a (5 mg/mL), fraction b (5 mg/mL) and CAOP (5 mg/mL), and the results are shown in Table 2.

### 3.7. Virtual Screening

PeptideRanker is a website for analyzing the potential physiological activity of peptides. The size of the score can be used to predict the potential of peptide physiological activity [24]. The BIOPEP - UWM™ database is a tool for studying the functions of bioactive peptides. It can detect repetition rates and mine potential antioxidant peptide sequences [25]. If repeated fragments are retrieved, more than 30%, then this shows that the peptide sequence has oxidation resistance. AnOxPePred is a tool used to evaluate the radical scavenging ability of peptides, providing a Scavenger score [26]. The Scavenger score is proportional to their ability to scavenge free radicals. To screen fraction b peptide sequences for cellular-level antioxidant potential, we utilized PeptideRanker, the BIOPEP-UWM™ database, and AnOxPePred. In total, 513 peptide sequences have a PeptideRanker score >0.6, 82 have a BIOPEP-UWM™ database match repetition frequency >30%, and 593 have a Scavenger score >0.4. Crucially, only 25 peptide sequences were confidently identified through mass spectrometry, with high credibility in the results (Table 3).

### 3.8. Electronic Properties and Keap1 Binding Capacity

We characterized the peptide’s electronic properties to elucidate its radical scavenging mechanism. Quantum mechanical computations suggest that the peptide’s antioxidant activity correlates with its electronic profile. Elevated HOMO energy (EHOMO) facilitates electron donation to electrophiles. Table 4 demonstrates EHOMO values.

**Figure 6:**
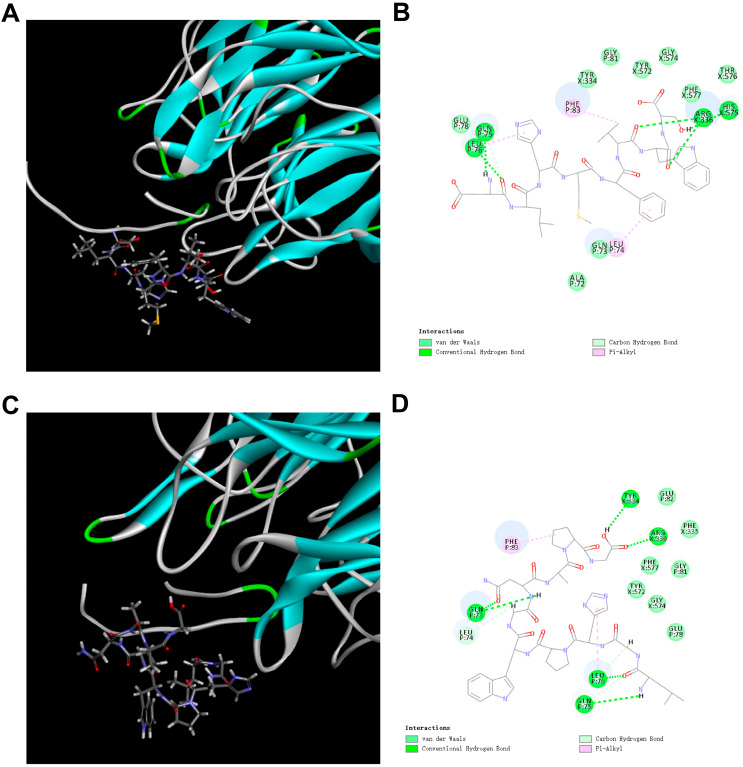
(**A**,**B**) Three-dimensional and two-dimensional molecular docking models of Keap1-DLHMFVWS. (**C**,**D**) Three-dimensional and two-dimensional molecular docking models of Keap1-LGHPWGNAPG. Interaction mode: 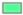
—van der Waals; 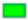
—conventional hydrogen bond; 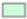
—carbon hydrogen bond; 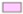
—pi–alkyl.

Peptide-Keap1 molecular docking was performed using CDOCKER, and the results are shown in Table 4. Huerta reports that TX6 can effectively bind to Keap1 and activate Nrf2, thus promoting the upstream components (ARE) and the expression of the oxidation reaction [27]. Molecular docking analysis identified TX6 (PubChem CID: 121488089) as the reference ligand (Keap1 binding energy: −17.74 kJ/mol), while DLHMFVWS (−62.81 kJ/mol) and LGHPWGNAPG (−57.43 kJ/mol) both exhibited higher binding affinities with Keap1 than TX6. TX6 interacts with amino acid residues of Keap1 through hydrogen bonds, van der Waals forces, and alkyl and pi–alkyl interactions (see the binding energy and interaction diagram of TX6 with Keap1 in the study by Ma et al. from the same laboratory [21]). DLHMFVWS and LGHPWGNAPG interact with the amino acid residues of Keap1 through the formation of van der Waals forces, hydrogen bonds, carbon–hydrogen bonds, and pi–alkyl interactions (Figure 6A–D). Therefore, these results indicate that DLHMFVWS and LGHPWGNAPG bind well to Keap1, and the stability of the DLHMFVWS and LGHPWGNAPG-Keap1 binding complexes is higher than that of the Keap1-TX6 complex.

## 4. Discussion

Reactive oxygen species (ROS) are oxidative free radicals generated during the body’s metabolic processes. Imbalances in ROS levels can disrupt the internal environment homeostasis [28]. Organisms rely on an antioxidant defense system comprising endogenous antioxidants like glutathione (GSH) [29] and antioxidant enzymes such as SOD, glutathione peroxidase (GSH-Px), and CAT to regulate ROS levels. This sustains ROS production–elimination equilibrium, preventing oxidative damage [30]. Oxidative stress occurs when elevated ROS levels impair normal cellular functions [31]. Moreover, oxidative stress can initiate a detrimental cycle wherein ROS-induced damage to biomolecules escalates ROS accumulation, leading to adverse health outcomes like organ dysfunction [32], immune dysregulation [33], inflammation [34], and tumorigenesis [35].

Numerous studies have demonstrated the antioxidant properties of various plant-derived natural compounds, including polyphenols, polysaccharides, and peptides [36,37,38]. Among these, peptides have garnered increasing interest due to their notable antioxidant activity. Antioxidant peptides sourced from food products are known for their high efficacy and low toxicity, making them promising candidates for applications in the food processing and nutraceutical industries [28]. Antioxidant peptides from animal and plant sources such as fish, milk, flaxseed, buckwheat and wheat have been used in the development of antioxidants for food and drugs [39,40,41,42,43]. In this study, CAOP has negligible toxicity to RAW264.7 cells and can protect against LPS-induced cell damage.

Previous studies have shown that intracellular amino acid metabolism is associated with inflammation and cellular iron death, e.g., cells under energetic stress are accompanied by a large amount of energy depletion leading to intracellular inflammation and cellular iron death [44]. Researchers have shown that glutathione metabolism is important for maintaining ROS homeostasis and intracellular redox homeostasis in cells. Oxidized glutathione (GSSG) is converted to reduced glutathione (GSH) in the presence of NADPH, which is itself a major natural antioxidant in animals, scavenging ROS and protecting cells from oxidative stress [45,46]. Cysteinyl, glycine and γ-glutamylcysteine, important metabolites in the glutathione pathway, are also important metabolites that can protect cells from oxidative stress, and γ-glutamylcysteine is a direct precursor of GSH, which has the capacity to detoxify ROS [47,48]. CAOP pretreatment elevated GSH, GSSG, and γ-glutamylcysteine concentrations in LPS-challenged RAW264.7 macrophages. Based on these results, it was hypothesized that CAOP has an anti-inflammatory effect by increasing glutathione metabolism levels and promoting GSH biosynthesis, thereby inhibiting cellular iron death and alleviating inflammation. In addition, we found that after LPS treatment, pantothenic acid and pantetheine 4′-phosphate were reduced. Pantetheine 4′-phosphate is an intermediate of pantothenic acid in the synthesis of CoA and pantothenic acid is an important precursor for the biosynthesis of CoA, which not only scavenges free radicals but also promotes the synthesis of phospholipids to help repair cellular plasma membranes from damage [49]. CoA not only scavenges free radicals but also promotes the synthesis of phospholipids to aid in cellular repair and protect the plasma membrane from damage; pantothenic acid also increases glutathione biosynthesis, thereby slowing apoptosis and cell damage [50]. We hypothesize that RAW264.7 cells promote the biosynthesis of pantothenic acid into coenzyme A (CoA) and then consume CoA to produce inflammatory and oxidative reactions. Pretreatment with CAOP reduces the consumption of pantothenic acid, further indicating that pretreatment with CAOP can alleviate cellular inflammation and oxidative stress to a certain extent.

Thiamine (vitamin B1) can regulate mitochondrial function and the metabolism of lipids, glucose, amino acids, and neurotransmitters. Thiamine (THA) is also an important water-soluble vitamin that mainly functions in the form of phosphorylated derivatives. In inflammatory conditions, thiamine interferes with the regulation of cellular oxidative metabolism [51,52,53]. Phosphorylated thiamine derivatives (ThMP/ThDP/ThTP) mediate bioenergetic metabolism, while their cationic species (T^+^) primarily functions as an antioxidant [54]. Thiamine depletion observed in LPS-stimulated versus untreated macrophages potentially contributes to anti-inflammatory and antioxidant responses in RAW264.7 cells. Thiamine, thiamine monophosphate, and pyridoxal 5′-phosphate were significantly elevated in RAW264.7 cells after CAOP pretreatment, suggesting that CAOP can ameliorate impaired oxidative energy metabolism through the thiamine pathway.

Glyoxylate and dicarboxylic acid metabolism are associated with oxidative reactions in vivo and can lead to abnormalities in the reactants and products of the TCA cycle. Thus, abnormalities in acetaldehyde and dicarboxylic acid metabolism indirectly affect intracellular energy metabolism. Citric acid has been shown to be an oxidative intermediate in metabolism, and citric acid reduces intracellular lipid peroxidation and inflammation levels [55]. An increase in citric acid promotes the tricarboxylic acid cycle, which in turn provides more energy for the body’s metabolism [56]. Citric acid levels were significantly reduced in the model injury group, suggesting an increase in energy expenditure, the acceleration of the tricarboxylic acid cycle and the excessive consumption of various reactants and products, as well as an effect on the associated glycolate and dicarboxylic acid metabolic pathways [57]. The content of citric acid and other related biomolecules increased in the CAOP pretreatment group, indicating that citric acid can be metabolized through the glycerate and dicarboxylic acid metabolic pathways, thereby restoring normal energy metabolism in RAW264.7 cells.

Electronic property analysis indicates that the radical scavenging activity of peptides is positively correlated with the energy level of the highest occupied molecular orbital (HOMO), where higher EHOMO values indicate stronger electron donor capabilities. The EHOMO values of SFRWQ, LGHPWGNAPG and DLHMFVWS are −6.992 eV, −5.5236 eV and −5.4507 eV, respectively. Keap1 functions as a negative regulator of Nrf2. A disruption of the Keap1-Nrf2 interaction enables Nrf2 release and nuclear translocation, leading to the activation of downstream antioxidant response elements [58]. Consequently, analyzing antioxidant compounds’ binding to Keap1 constitutes an effective approach for assessing their capacity to enhance endogenous antioxidant defenses. Although SFRWQ exhibits optimal HOMO and LUMO energy, its established biological activity has been reported elsewhere [21]. Critically, DLHMFVWS and LGHPWGNAPG demonstrate superior Keap1-binding affinity despite moderate electronic profiles. The complexes formed between DLHMFVWS and LGHPWGNAPG and Keap1 are more stable than the TX6 and SFRWQ-Keap1 complex, indicating that they also possess significant advantages in regulating redox homeostasis.

This study has the following limitations. First, the in vitro experimental results indicate that CAOP has antioxidant and anti-inflammatory effects, but future studies in animal models are needed to validate the efficacy of these protective effects at the systemic level. In subsequent experiments, it is necessary to synthesize antioxidant peptides such as DLHMFVWS and LGHPWGNAPG to investigate their intracellular activity and mechanisms of action, thereby providing theoretical support for the industrial production of peptide substances from Cyperus.

## 5. Conclusions

CAOP, an antioxidant peptide derived from Cyperus, augments cellular antioxidant defenses by elevating SOD and CAT activities in RAW264.7 macrophages while suppressing IL-6 and TNF-α secretion and attenuating phagocytosis in LPS-stimulated cells, demonstrating dual antioxidant and anti-inflammatory functionality. Metabolomics analysis revealed the protective mechanism of CAOP, which is achieved by upregulating glutathione metabolism pathways, glyceraldehyde and dicarboxylic acid metabolism pathways, pantothenic acid and coenzyme A biosynthesis metabolism pathways, and thiamine metabolism pathways while inhibiting ferroptosis. Peptideomics and molecular docking data identified two core bioactive peptides (DLHMFVWS and LGHPWGNAPG), which bind to Keap1 to activate the Nrf2-Keap1 pathway.

In summary, CAOP exerts dual antioxidant and anti-inflammatory effects through multiple metabolic pathways and mechanisms. These findings establish CAOP as a promising therapeutic candidate with significant translational potential for practical medical applications, including as a natural peptide drug or supplement for the pharmacological treatment of metabolic inflammatory diseases such as diabetes and non-alcoholic fatty liver disease, dietary therapy targeting chronic inflammation in metabolic syndrome through functional food components, and preventive medicine targeting antioxidant stress-related diseases through multi-pathway interventions, thereby providing innovative therapeutic and nutritional strategies for metabolic health management.

## Figures and Tables

**Table 1:** Liquid chromatography gradient elution program.

Time	Buffer A	Buffer B
0	96%	4%
3	92%	8%
89	72%	28%
109	60%	40%
110	5%	95%
120	5%	95%

**Table 2:** DPPH radical scavenging activity.

	CAOP	Fractions a	Fractions b
DPPH clearance rate	88.69%	81.26%	90.63%

**Table 3:** Component scoring table.

	Peptide Sequence	Theoretical Mass-to-Charge Ratio	PeptideRanker Scores	Rate of Repetition	Scavenger Score
1	LWGR	530.6243	0.912846	75.00%	0.49713
2	GHPWG	552.599	0.945357	60.00%	0.61598
3	SFRWQ	722.799	0.891748	40.00%	0.43137
4	TLGHPWG	766.8586	0.608396	42.86%	0.58287
5	DLHMFVWS	1034.1933	0.613035	37.50%	0.41534
6	FAY	399.4395	0.871389	33.33%	0.46884
7	YLW	480.5595	0.948405	33.33%	0.55369
8	WSY	454.4795	0.82987	33.33%	0.51467
9	FHL	415.4895	0.88902	33.33%	0.47922
10	IIWYTL	807.9738	0.707751	33.33%	0.50076
11	WLH	454.5295	0.904459	33.33%	0.5521
12	IWY	480.5595	0.886712	33.33%	0.54823
13	VWF	450.5395	0.975754	33.33%	0.47109
14	WGI	374.4395	0.941714	33.33%	0.48014
15	QPRYLP	772.8938	0.76001	33.33%	0.50282
16	IPW	414.4995	0.959086	33.33%	0.49862
17	AYF	399.4395	0.915352	33.33%	0.48733
18	LAW	388.4595	0.879554	33.33%	0.44419
19	WGW	447.4995	0.996392	33.33%	0.57219
20	FVW	450.5395	0.974429	33.33%	0.4388
21	WIY	480.5595	0.884482	33.33%	0.52127
22	MYL	425.5395	0.816343	33.33%	0.49952
23	TWL	418.4895	0.776305	33.33%	0.45991
24	IVW	416.5195	0.603901	33.33%	0.41524
25	LGHPWGNAPG	1005.1028	0.713675	30.00%	0.51702

**Table 4:** Tertiary structure of peptides: HOMO and LUMO, molecular energy and docking energy with Keap1.

	Sequence of Peptide	HOMO (ev)	LUMO (ev)	Molecular Energy (a.u.)	-ICE
1	DLHMFVWS	−5.4507	−1.1325	−3809.8757	62.8072
2	LGHPWGNAPG	−5.5236	−0.6152	−3459.8023	57.4345
3	SFRWQ	−6.9920	−0.1101	−2472.8600	49.3525
4	LWGR	−6.9546	0.1952	−1789.8200	47.4082
5	IIWYTL	−5.8627	−0.5208	−2696.6552	43.0278
6	QPRYLP	−5.7266	−1.1627	−2629.4169	40.4706
7	TLGHPWG	−5.5236	−0.6152	−2625.8286	38.9967
8	AYF	−6.0262	−0.4726	−1355.3300	33.4841
9	FVW	−5.5519	−0.4343	−1490.2817	32.4883
10	YLW	−5.6167	−0.4707	−1604.7700	32.4435
11	WGW	−5.4719	−0.4188	−1503.8900	31.9292
12	FAY	−6.1293	−0.7725	−1355.3300	29.1102
13	WSY	−5.2632	−0.7638	−1562.0300	28.9494
14	IVW	−0.7721	3.4173	−1368.4686	28.8689
15	VWF	−5.5876	−0.4090	−1490.2800	29.5592
16	GHPWG	−6.9375	0.1404	−1899.5600	27.8538
17	LAW	−5.5522	−0.4441	−1298.5900	27.9754
18	MYL	−5.5745	−0.8579	−1719.0041	27.2575
19	TWL	−5.9503	−0.7564	−1413.0840	27.0853
20	WGI	−5.4961	−0.8079	−1259.2700	26.2893
21	IPW	−5.4243	−0.5412	−1375.9600	26.2193
22	IWY	−5.6442	−0.4528	−1604.7700	26.1046
23	WIY	−5.3859	−0.8465	1604.7711	25.0357
24	WLH	−5.2948	−1.1453	−1523.5100	23.5987
25	FHL	−6.5590	−0.7031	−1392.0000	19.8777
	TX6				17.7358

## Data Availability

The original contributions presented in this study are included in the article. Further inquiries can be directed to the corresponding author.

## Abbreviations

The following abbreviations are used in this manuscript:

SOD	Superoxide dismutase
CAOP	Cyperus peptide
CAT	Catalase
β-actin	Beta-actin
TNF-α	Tumor necrosis factor-alpha
IL-6	Interleukin-6
GSH	Reduced glutathione
GSSG	Oxidized glutathione
Elisa	Enzyme-linked immunosorbent assay
